# Lethal and Sublethal Effects of the Novel *cis*-Nitromethylene Neonicotinoid Cycloxaprid on the Green Peach Aphid, *Myzus persicae* (Sulzer) (Hemiptera: Aphididae)

**DOI:** 10.3390/toxics14010030

**Published:** 2025-12-26

**Authors:** Junshu Zhu, Li Wang, Zongyin Cui, Weiling Huang, Qinqin Wang, Wenjie Wang, Qingjie Yang, Changhui Rui, Li Cui

**Affiliations:** 1 Key Laboratory of Integrated Pest Management in Crops, Institute of Plant Protection, Chinese Academy of Agricultural Sciences, Ministry of Agriculture and Rural Affairs, Beijing 100193, China; 15765987687@163.com (J.Z.); wlhenanau2022@163.com (L.W.); zcui@stu.scau.edu.cn (Z.C.); wl18210335803@163.com (W.H.); wangqqhau@163.com (Q.W.); wz02040118@163.com (W.W.); yang_qingjie@163.com (Q.Y.); chrui@ippcaas.cn (C.R.); 2 College of Plant Protection, Fujian Agriculture and Forestry University, Fuzhou 350002, China; 3 College of Plant Protection, Henan Agricultural University, Zhengzhou 450002, China

**Keywords:** green peach aphid, cycloxaprid, lethal effect, life table

## Abstract

*Myzus persicae* is a worldwide insect pest with high resistance to many traditional insecticides. Cycloxaprid, a novel cis-configuration neonicotinoid insecticide, is effective in controlling neonicotinoid-resistant insect pests. Lethal and sublethal effects of cycloxaprid on *M. persicae* were conducted in this study. Results showed that cycloxaprid had higher toxicity to the laboratory and field resistant *M. persicae* than imidacloprid. Because of the resistance, imidacloprid showed lower control efficacy (<60%) against *M. persicae*, which falls short of the efficacy required for practical agricultural management. However, cycloxaprid exhibited higher control efficacies (>84.79%) against *M. persicae* in the field. In addition, in order to quantify the sublethal impacts of cycloxaprid, we conducted a life table analysis on *M. persicae*. When resistant *M. persicae* was treated with LC_25_ of cycloxaprid or imidacloprid, the longevity and fecundity of F_1_ adults were significantly decreased. Meanwhile, the intrinsic rate of increase (*r_m_*), finite rate of increase (*λ*) and net reproduction rate (*R_i_*) of F1 generation *M. persicae* were reduced in cycloxaprid and imidacloprid treatments. Therefore, cycloxaprid shows high potential as a candidate insecticide for managing imidacloprid-resistant *M. persicae*. Importantly, our laboratory data indicate that exposure to its low sublethal concentration (LC_25_) inhibits population growth parameters, suggesting a low risk of inducing pest resurgence under such conditions.

## 1. Introduction

The green peach aphid (GPA), *Myzus persicae* (Sulzer) (Hemiptera: Aphididae), is a highly destructive agricultural pest with a worldwide distribution, attacking hundreds of species from 40 plant families [1]. As a sap-feeding pest, *M. persicae* could cause severe damage by direct feeding, promoting growth of sooty mold, blocking photosynthesis and transmitting plant viruses [2]. Although the widespread use of chemical controls has led to the evolution of resistance to at least 80 different active ingredients in *M. persicae*, making this species one of the most strongly and widely resistant insect worldwide, at present, insecticides remain the primary tool for control of *M. persicae* worldwide [3]. Among these insecticides, neonicotinoids are the leading products, which have high selectivity and affinity for nicotinic acetylcholine receptors in insect central nervous system [4]. This resistance crisis has been exacerbated by the extensive and sustained application of key trans-nitromethylene neonicotinoids, including imidacloprid (2350-flod), thiamethoxam (270-fold), clothianidin (3013-fold) and thiacloprid (>2500-fold) [3,5,6]. The increasing number of resistance events mean there are very few effective insecticides remaining to control *M. persicae*. Therefore, a new insecticide with novel mechanisms should be introduced to against the resistant *M. persicae*.

Cycloxaprid belongs to a novel subclass of neonicotinoids featuring a cis-configured nitromethylene group, in direct contrast to the trans-configuration characteristic of other commercial neonicotinoids [7]. The novel chemical structure endows it different binding site and wide insecticidal spectrum [8,9]. Cycloxaprid has been reported to exhibit high insecticidal activity against many sap-sucking insect pests, such as *Nilaparvata lugens* [10,11], *Apolygus lucorum* [8], *Aphis gossypii* [12] and *Bemisia tabaci* [13]. Furthermore, cycloxaprid retains efficacy against pest populations that have developed resistance to established neonicotinoids like imidacloprid [8,10,12,14]. For example, cycloxaprid shows 2-fold higher activity against imidacloprid-resistant *Bemisia tabaci* when compared with imidacloprid [13]. In addition, it has been reported that cycloxaprid showed high insecticidal activity, low cross-resistance and different binding properties on insect nAChRs. Therefore, cycloxaprid is a potential insecticide for the control of insect pests, especially ones with high resistance to neonicotinoids [11].

A complete assessment of a novel insecticide requires evaluation beyond lethal toxicity to include its potential sublethal effects. Sublethal effects refer to the physiological or behavioral effects of pests exposed to low or sublethal concentrations of toxic substances [15]. Due to the degrade of pesticides after initial applications to crops, insect pests exposure to sublethal concentrations of pesticides in agro-ecosystems is a common phenomenon [16,17]. Sublethal concentrations of insecticide can’t kill the entire population of insects but exert physiological or behavioral induction on individuals, including decreased pests developmental time or increased adult longevity and reproduction [18,19,20,21]. However, these sublethal impairments carry significant ecological and resistance management risks, such as accelerated evolution of resistance and potential pest resurgence [19,22,23,24]. Therefore, a thorough evaluation of a pesticide’s total population-level impact is critical for informed insecticide selection and sustainable pest management. In addition, the sublethal effects of cycloxaprid on *M. persicae* have not been reported. Therefore, we used age-stage life table analysis to evaluate the sublethal effects of cycloxaprid on field *M. persicae*. This approach was adopted to generate essential data for resistance management strategies and to comprehensively assess the efficacy and potential risks associated with this novel insecticide.

## 2. Materials and Methods

### 2.1. M. persicae Strains

The susceptible strain *M. persicae* was reared in the laboratory and the field resistant strain of *M. persicae* was obtained from the experimental peach tree in Shandong Province, China. Two strains were maintained on radish seedlings (*Raphanus sativus* L.) without exposure to insecticides, under a 25 ± 1 °C, 60 ± 10% humidity with a L16:D8 h photoperiod.

### 2.2. Insecticides and Chemicals

Cycloxaprid (97%) and cycloxaprid 25% WP were provided by Shanghai Shengnong Pesticide Co., Ltd., Shanghai, China. Imidacloprid (95.5%) was provided by Jiangsu Kesheng Co., Ltd., Yancheng, China. Imidacloprid 10% WP was obtained from Nanjing Red Sun Co., Ltd., Nanjing, China. Triton X-100 and dimethylsulfoxide (DMSO) were obtained from Beijing chemical reagent Co., Ltd., Beijing, China.

### 2.3. Laboratory Trials of Cycloxaprid and Imidacloprid Toxicity

The toxicity of imidacloprid and cycloxaprid to *M. persicae* was determined using leaf-dipping method [14]. The concentration ranges for imidacloprid (susceptible strain: 1.56–25 mg/L; field strain: 25–400 mg/L) and cycloxaprid (susceptible strain: 1.25–20 mg/L; field strain: 5–80 mg/L) were chosen to encompass the anticipated LC_50_ values. A geometric series of five concentrations was prepared for each insecticide by serial dilution with an aqueous solution of Triton X-100 (0.05% *w*/*v*). Individual *raphanus sativus* leaves with about 50 adult *M. persicae* were dipped in the diluted solutions for 10 s or in to 0.05% Triton X-100 aqueous solution (as the control group). After drying, the leaves were transferred to 90 mm petri dishes containing filter paper moistened with deionized water. Then, *M. persicae* were reared at 27 ± 1 °C, 60 ± 10% humidity and a 14:10 h light:dark photoperiod. After 24 h, the mortality was observed under a microscope. Mortality was recorded when *M. persicae* exhibited no signs of movement or response to physical stimuli. Bioassays for each concentration consisted of 4 replicates. Laboratory toxicity tests were conducted on adult *M. persicae*.

### 2.4. Field Control Using Cycloxaprid and Imidacloprid

The field control effects of cycloxaprid on *M. persicae* were evaluated in Linyi city, Shandong Province (Coordinates: 35.25° N, 118.55° E). The active ingredient concentrations evaluated in the study were 100 mg·L^−1^ for cycloxaprid and 133.3 mg·L^−1^ for imidacloprid. The electric knapsack sprayer T-HS 16D (Shandong Agricultural Pharmaceutical Equipment Co., Ltd., Laiyang, China) was used to spray insecticide. In order to reduce the impact of other insecticides, no insecticide spray was applied until the scheduled insecticide application. Each plot contained two peach trees, and 10 leaves containing *M. persicae* were randomly selected from each plot. Control plots received an application of water only. Each treatment consisted of three repetitions. Bioassays were arranged in a complete randomized block design. The numbers of living *M. persicae* were investigated 3, 7 and 14 days after application. Within each experimental plot (treatment unit), 10 leaves with moderate and representative aphid infestation were randomly selected from the mid-canopy of the two peach trees (5 leaves per tree). To ensure randomness and avoid bias, leaves were selected from all four cardinal directions around the canopy. All live *M. persicae* (both nymphs and adults) on the upper and lower surfaces of each selected leaf were counted in situ using a hand-held magnifying lens, without disturbing the insects or removing the leaves. *M. persicae* were considered dead if they showed no movement and no response to gentle physical stimulation. Field efficacy trials were conducted primarily on adults and nymph populations of *M. persicae.*

### 2.5. Laboratory-Measured Sublethal Effects of Cycloxaprid and Imidacloprid

Sublethal effects experiments were conducted on adult *M. persicae*, with F1-generation phenotypes assessed to evaluate sublethal impacts. The sublethal effects of cycloxaprid and imidacloprid on *M. persicae* were evaluated using the concentration of LC_25_. In order to mimic the lower concentrations of pesticides that may occur in the field after initial insecticide application, LC_25_ concentrations of imidacloprid and cycloxaprid were used in this study. At least 300 adults were treated with LC_25_ of imidacloprid or cycloxaprid by the leaf dipping method. Control adults were treated with distilled water (containing 0.05% (*w*/*v*) Triton X-100). Mortality was calculated at 24 h after treatment. The surviving *M. persicae* were transferred to a separated petri dish with fresh cabbage leaves. After 12 h, the offspring produced by the F_0_ adults were collected and used as the F_1_ generation in the life table experiment. At least 100 neonate nymphs of each group were tested individually. Population parameters such as development time of each stage, total longevity, survival and fecundity of F_1_ generation were recorded every day. Newly born nymphs were recorded and removed daily. The longevity of *M. persicae* and the number of nymphs produced per adult were recorded until it adult died. All experiments were carried out at 27 ± 1 °C, 60 ± 5% RH and 16:8 (L:D) h photoperiod.

### 2.6. Statistical Analysis

Statistical software SPSS 13.0 (SPSS Inc., Chicago, IL, USA) was used to calculate the sublethal concentration (LC_25_), median lethal concentration (LC_50_) and their 95% confidence intervals (CIs). In the field experiment, the efficacies of insecticides were transformed using arcsine square root transformation, expressed in degrees as (180/π) × arcsin ($\sqrt{x}$), prior to statistical analysis. Data were statistically analyzed using one-way analysis of variance (ANOVA) followed by Fisher’s LSD tests and *t*-tests (α = 0.05). The daily fecundity, longevity and survivorship of *M. persicae* individuals were analyzed using the theory and method of age stage and a two-sex life table. The Age-specific Survival Rate (*l_x_*), Age-specific Fecundity (*m_x_*), Age-specific Maternity (*l_x_m_x_*), Reproductive Value (*v_x_*), Age-stage Survival Rate (*s_xj_*), Age-stage Life Expectancy (*e_xj_*), Age-specific Life Expectancy (*e_x_*), Average Population (APOP), Total Population (TPOP), Intrinsic Rate of Increase (*r_m_*), Finite Rate of Increase (*λ*), Net Reproduction Rate (*R_i_*) and Mean Generation Time (*T*) were calculated. In the TWOSEX-Mschart computer program, bootstrapping methods (100,000 repetitions used in the bootstrapping procedure) were used to evaluate mean values, standard errors and significant differences. The data were constructed using Origin 7.0 software (OriginLab Corporaton, Northampton, MA, USA).

## 3. Results

### 3.1. Toxicity of Cycloxaprid and Imidacloprid Against Different Strains of M. persicae

The LC_50_ and LC_25_ values of cycloxaprid and imidacloprid against laboratory susceptible and field resistant strains of *M. persicae* were shown in Table 1. The results showed that cycloxaprid showed higher toxicity against both strains of *M. persicae* than imidacloprid. The LC_50_ values of cycloxaprid were 2.28 and 8.31 mg·L^−1^, respectively for the laboratory susceptible and field resistant strains of *M. persicae*. However, the LC_50_ values of imidacloprid were up to 5.99 and 90.43 mg·L^−1^, respectively for two strains. A significant decrease in susceptibility to imidacloprid was observed in the field strain of *M. persicae*, and this field strain showed moderate resistance to imidacloprid compared with the laboratory susceptible strain. The LC_25_ values of cycloxaprid and imidacloprid were 3.05 and 25.28 mg·L^−1^, respectively for the field strain, and these concentrations were used as sublethal concentrations for subsequent experiments.

### 3.2. The Control Efficacies of Cycloxaprid and Imidacloprid Against M. persicae in the Field

Table 2 showed the control efficacies of cycloxaprid and imidacloprid against *M. persicae* in the field. The results demonstrated that cycloxaprid had better control efficacies against *M. persicae* than imidacloprid. The efficacy of cycloxaprid was up to 93.33% after 3 days treatment at the concentration of 100 mg·L^−1^. Moreover, cycloxaprid showed long persistence, and the efficacy was 84.79% after 14 days treatment, whereas imidacloprid showed lower control efficacy (<60%) against *M. persicae*.

### 3.3. Sublethal Effects of Cycloxaprid and Imidacloprid on F_0_ Generation of M. persicae

**Figure 1:**
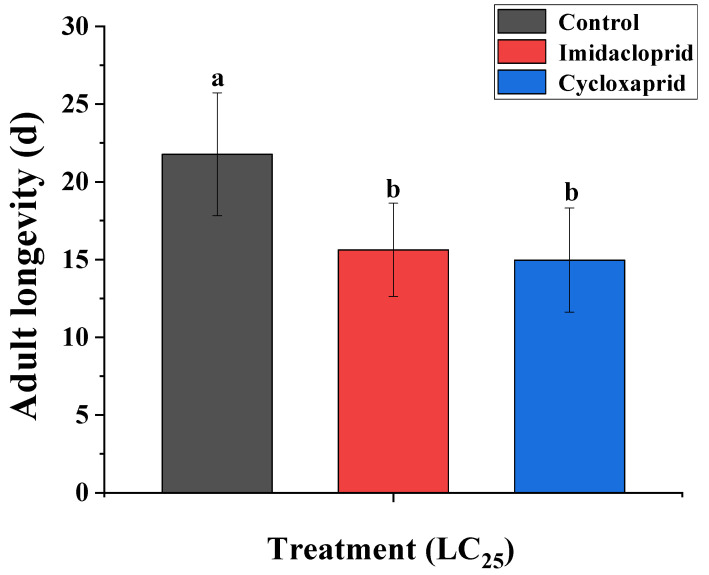
Sublethal effects of cycloxaprid and imidacloprid on adult longevity for the F_0_ generation of *M. persicae*. Error bars represent 95% confidence intervals (95% CI). Different letters suggest significant differences between groups and were determined by Student *t*-test using SAS 9.2 (*p* < 0.05).

**Figure 2:**
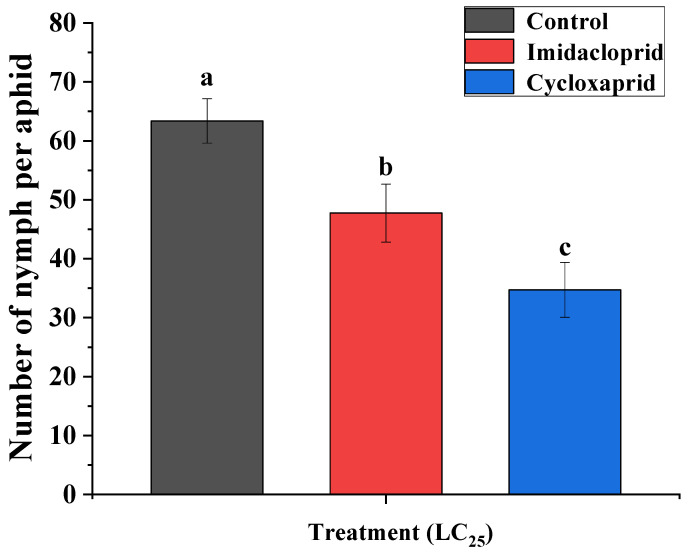
Sublethal effects of cycloxaprid and imidacloprid on adult fecundity for the F_0_ generation of *M. persicae*. Error bars represent 95% confidence intervals (95% CI). Different letters suggest significant differences between groups and were determined by Student *t*-test using SAS 9.2 (*p* < 0.05).

The result suggested that the LC_25_ values of cycloxaprid and imidacloprid to the field strain of *M. persicae* were 3.05 and 25.28 mg·L^−1^, respectively. Figure 1 and Figure 2 showed the sublethal effects of cycloxaprid and imidacloprid on adult longevity and fecundity for the F_0_ generation of *M. persicae*. Compared with the control group, LC_25_ of cycloxaprid and imidacloprid significantly decreased the longevity and fecundity of F_0_ generation of *M. persicae*. Moreover, Figure 2 showed that cycloxaprid had a more significant inhibition on the fecundity of F_0_ generation of *M. persicae* than imidacloprid.

### 3.4. Intergenerational Effects of Cycloxaprid and Imidacloprid on the F_1_ Generation of M. persicae

The sublethal effects of imidacloprid and cycloxaprid on the fecundity, longevity and development time of *M. persicae* in the F_1_ generation were also investigated (Table 3). Cycloxaprid significantly prolonged the developmental time of 1st instar 2nd instar nymph, while the developmental time of adults were shortened by cycloxaprid and imidacloprid. Moreover, cycloxaprid and imidacloprid significantly decreased the fecundity and increased the total preoviposition period (TPOP) of *M. persicae*. Therefore, F_1_-generation *M. persicae* treated with LC_25_ of cycloxaprid and imidacloprid generated lower offspring than the control aphids.

The bootstrap methods of life table were used to estimate the transgenerational effects of sublethal cycloxaprid and imidacloprid on population dynamics. The *r_m_*, *λ*, *R_i_* and *T* were calculated and analyzed (Table 4). Significant differences were observed in the cycloxaprid- and imidacloprid-treated groups compared with the control. LC_25_ of cycloxaprid and imidacloprid significantly decreased the net reproductive rate, intrinsic rate of increase and finite rate of increase (*λ*), while the mean generation time of F_1_ generation was increased by sublethal concentration of cycloxaprid.

**Figure 3:**
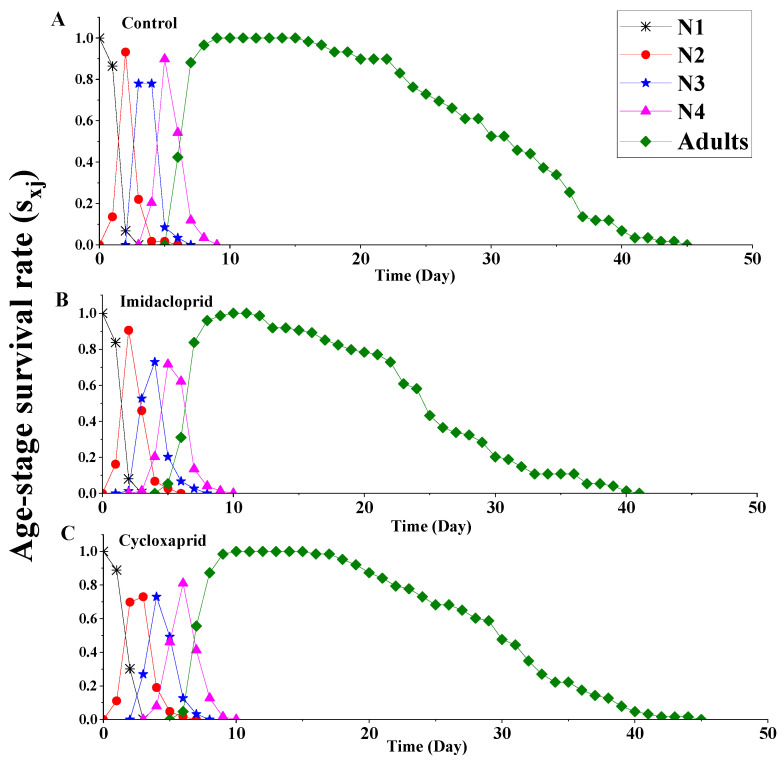
Age-stage survival rate (*S_xj_*) of the F_1_ generation of *M. persicae* descending from F_0_ individuals exposed to LC_25_ of cycloxaprid and imidacloprid. (**A**) it represents the control group; (**B**) it represents the imidacloprid treatment group; (**C**) it represents the cycloxaprid treatment group.

Age-stage survival rate (*S_xj_*) curves represent the probability that a newborn nymph will survive to age x and stage j after treatment by imidacloprid or cycloxaprid at LC_25_ concentration (Figure 3). The adult stage became shorter if the F_0_ adults were treated with LC_25_ of imidacloprid. The maximal survival rate for second-instar (N2), third-instar (N3) and fourth-instar (N4) nymphs was lower in the cycloxaprid-treated group than in the control group. At the same time, the maximal survival rate of adults was reduced by exposure to the sublethal concentration of imidacloprid.

**Figure 4:**
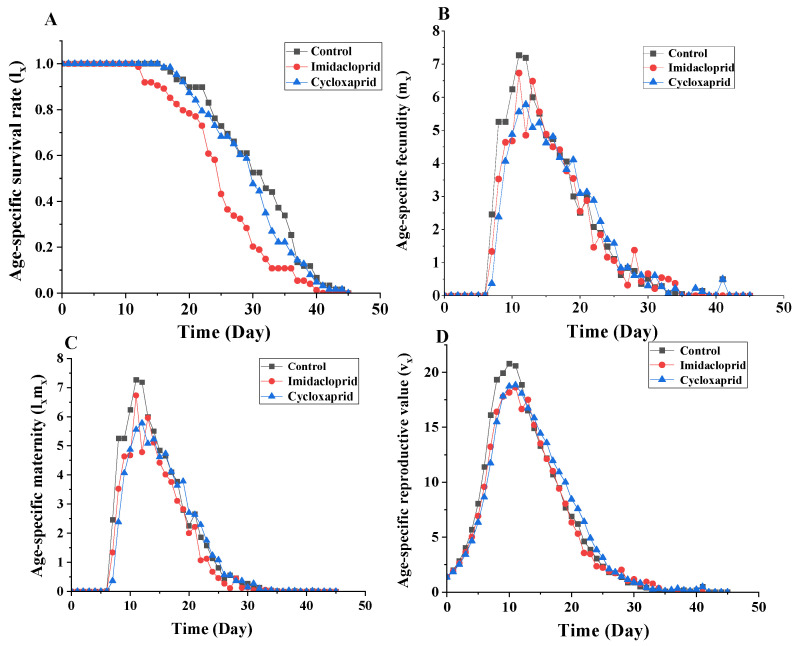
Age-specific survival rate (*l_x_*) (**A**), Age-specific fecundity (*m_x_*) (**B**), Age-specific maternity (*l_x_m_x_*) (**C**), and Age-specific reproductive value (*v_x_*) (**D**) of the F_1_ generation of *M. persicae* descending from F_0_ individuals exposed to LC_25_ of cycloxaprid and imidacloprid.

The age-specific survival rate (*l_x_*) curve shows the probability that a newborn nymph will survive to age x, and the curve is a simplified overview of the survival history, regardless of the different developmental stage. The age-specific survival rate of imidacloprid-treated *M. persicae* was significantly lower than that of the control group (Figure 4A). Based on the age-specific fecundity (*m_x_*) curve, the highest peak in control *M. persicae* occurred at the age of 12 days (Figure 4B). However, the age-specific fecundity of cycloxaprid-treated *M. persicae* was lower than that of the control group at the age of 6 to 15 days. The *l_x_m_x_* value changed depending on *l_x_* and *m_x_*, the maximum *l_x_m_x_* values were 7.27, 6.73 and 5.78 for the control, imidacloprid-treated and cycloxaprid-treated group, respectively (Figure 4C). Cycloxaprid and imidacloprid could reduce the age-specific reproductive value (*v_x_*) of F_1_-generation *M. persicae* (Figure 4D). The maximum vx values of cycloxaprid-treated (18.83 at the age of 11 days) and imidacloprid-treated (18.63 at the age of 11 days) group were lower than that of the control group (20.78 at the age of 11 days).

**Figure 5:**
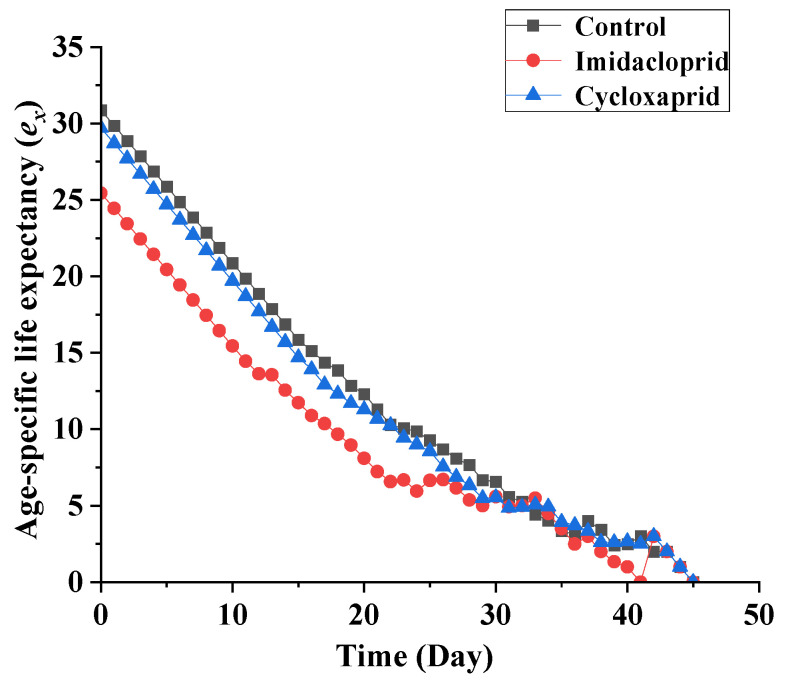
Age-specific life expectancy (*e_x_*) of the F_1_ generation of *M. persicae* descending from F_0_ individuals exposed to LC_25_ of cycloxaprid and imidacloprid.

**Figure 6:**
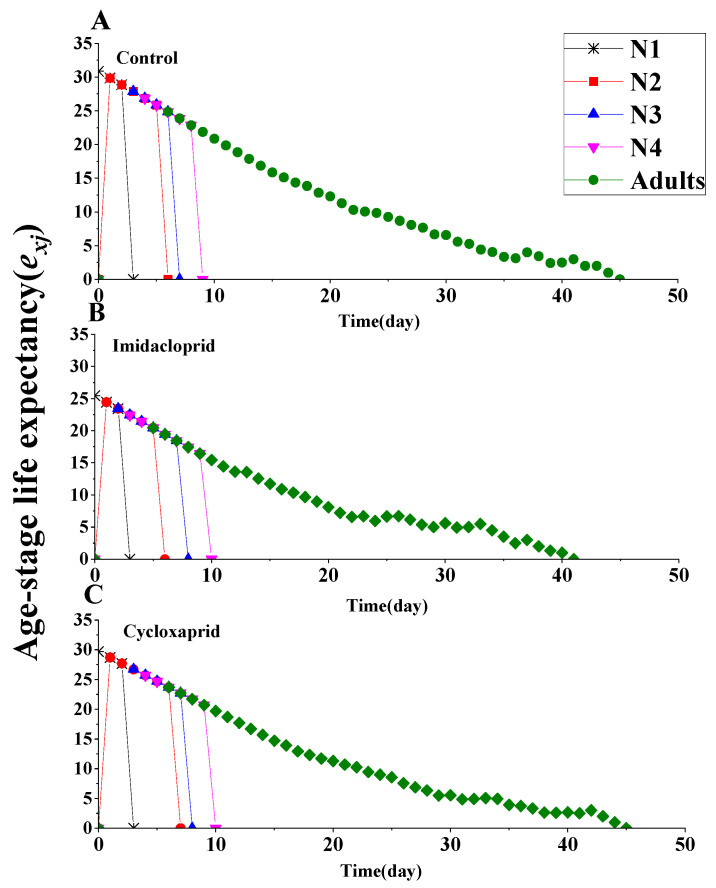
Age-stage life expectancy (*e_xj_*) of the F_1_ generation of *M. persicae* descending from F_0_ individuals exposed to LC_25_ of cycloxaprid and imidacloprid. (**A**) it represents the control group; (**B**) it represents the imidacloprid treatment group; (**C**) it represents the cycloxaprid treatment group.

Age-specific life expectancy (*e_x_*) curve indicates that the life expectancy of an individual of age x. According to Figure 5, imidacloprid and cycloxaprid reduced the life expectancy of F_1_ generation *M. persicae*. Age-stage life expectancy (*e_xj_*) curve indicates the time that an individual of age x and stage y is expected to live (Figure 6). The life expectancy of first-instar nymphs, second-instar nymphs, third-instar nymphs, fourth-instar nymphs and adults were 30.86, 29.86, 27.86, 26.86 and 24.86 days, respectively, for the control group, while these values were only 25.45, 24.45, 23.45, 22.45 and 20.45 days, respectively, for imidacloprid-treated *M. persicae*, and were 29.70, 28.70, 26.70, 25.70 and 23.70, respectively, for cycloxaprid-treated *M. persicae* (Figure 6).

## 4. Discussion

The widespread usage of chemicals has contributed to insecticide resistance [25,26], and even worse, since 2010, the resistance of *M. persicae* to neonicotinoid insecticides increased dramatically [27]. Imidacloprid is a representative neonicotinoid and it is active on a broad range of sap-feeding Insects, including *M. persicae* [28]. However, there were 569 documented cases of imidacloprid resistance in 2021, 99 of those cases involving *M. persicae* [27]. Similarly, our study confirmed a moderate level of resistance to imidacloprid (RR = 15.97) in the field population of *M. persicae* from Linyi city, Shandong Province. Moreover, the field control efficiency of imidacloprid against *M. persicae* was lower than 55.2% at 133.33 mg·L^−1^. This result obviously cannot satisfy the demand of agricultural production practices. The emergence of multi-insecticide resistance in *Myzus persicae* has posed a significant challenge to its sustainable management [29,30]. Thus, faced with the fact that there are increasing cases of pest resistance to neonicotinoids, especially for sap-feeding pests such as *M. persicae*, there is a requirement for the use of new insecticidal chemistries with novel mechanisms to maintain the effective pest control efficacy and to delay the resistance development and to reduce the negative impacts to the environment [27,31,32].

Cycloxaprid was the first cis-nitromethylene neonicotinoid insecticide, whereas in all other neonicotinoid insecticides, the cyano or nitro cyano group is in the trans-configuration [33]. Cycloxaprid acts on insect nAChRs as other commercial neonicotinoid insecticides such as imidacloprid, but it only had partially overlapped binding sites with imidacloprid in the insect central nervous system [11]. Therefore, cycloxaprid not only showed high toxicity to Hemipteran insect pests such as *Aphis craccivora*, *A. gossypii*, *Bemisia tabaci* and *N. lugens*, but also was seldom affected by the resistance to other neonicotinoid insecticides in pests, such as imidacloprid resistance in *A. gossypii*, *B. tabaci* and *N. lugens* [10,11,12,13]. Similarly, our results suggested that the control effect of cycloxaprid was more than 90% at 100 mg·L^−1^ within 7 days in field. It is obviously superior to imidacloprid (Table 2). In addition, the field results are consistent with those obtained in the laboratory (Table 1). Briefly, compared with imidacloprid, cycloxaprid showed higher toxicity and control efficacy to *M. persicae* in laboratory in the field. Therefore, cycloxaprid is regarded as a promising insecticide against *M. persicae*.

Sublethal pesticide effects on pests typically result in changes to biological characteristics such as growth, fecundity and lifespan [34,35,36]. *M. persicae* will also be exposed to sublethal concentrations of cycloxaprid in the field due to uncontrollable factors such as degradation. The accurate assessment of the overall insecticide efficacy could be obtained by estimating either lethal or sublethal effects [37]. Therefore, we not only investigated the toxicity and field control effect, but also studied the sublethal effects of cycloxaprid on *M. persicae*. The incorrect use of insecticides may lead to pest outbreaks [38]. For example, sublethal concentrations of imidacloprid, thiamethoxam, acetamiprid and lambda-cyhalothrin exposure increased pest fecundity in the parent generation or their offspring generations [18,24,39,40,41]. The LC_25_ dose is not only useful for mimicking low field concentrations, but doses below LC_30_ are also widely considered safe for natural enemies in integrated pest management (IPM) programs. However, our study demonstrated that LC_25_ of cycloxaprid significantly reduced the longevity and fecundity of F0 and F_1_ generations of *M. persicae* adults. Therefore, the intrinsic rate of increase (*r_m_*), net reproductive rate (*R_i_*) and finite rate of increase (*λ*) of the F_1_ generation *M. persicae* were significantly inhibited by sublethal concentration (LC_25_) of cycloxaprid. In addition, cycloxaprid significantly prolonged the developmental time of 1st instar and 2nd instar nymphs, thereby increasing the mean generation time of F_1_ generations of *M. persicae*. Compared with the control and imidacloprid-treated group, cycloxaprid had the highest inhibition on the age-specific fecundity of *M. persicae*. So, we concluded that a sublethal dose of cycloxaprid is likely to inhibit rather than promote the population growth of *M. persicae*. Similarly, it has been reported that the longevity and fertility of cotton aphid adults was significantly decreased when treated with LC_10_ and LC_40_ of cycloxaprid [42]. It is important to note that while the observed sublethal effects (e.g., reduced *r_m_*, *R_i_* and *λ*) were statistically significant in our controlled laboratory study, their exact magnitude and practical impact in the agricultural fields remain to be quantified. Field populations are exposed to spatially and temporally variable insecticide residues, and their response may be influenced by additional biotic and abiotic factors not present in the laboratory. Thus, further field-based studies monitoring population dynamics after sublethal exposure are warranted to confirm the ecological implications of these findings and to validate cycloxaprid long-term management potential against *M. persicae*.

In conclusion, cycloxaprid is identified as a promising neonicotinoid insecticide for the management of *M. persicae*. It is highly toxic against the imidacloprid-resistant *M. persicae*. In addition, the sublethal concentration of cycloxaprid can reduce the fecundity rate of *M. persicae*. Therefore, based on our laboratory data, a resurgence in the *M. persicae* population is less likely to be induced by exposure to low sublethal concentrations of cycloxaprid, as compared to some other insecticides that have shown hormetic effects. However, the definitive assessment of this risk under complex field conditions, where residual concentrations are dynamic and heterogeneous, requires further investigation. Given its unique cis-nitromethylene structure and partially non-overlapping binding site, cycloxaprid should be rotated with insecticides from entirely different chemical classes to mitigate selection pressure and delay the evolution of broad-spectrum resistance. Cycloxaprid is an excellent candidate to replace or alternate with conventional neonicotinoids in regions where high resistance to the latter has been confirmed, as shown in this study. This study will contribute to the overall assessment of cycloxaprid on *M. persicae* and provide data support for the integrated control of *M. persicae*.

## 5. Conclusions

This study demonstrates that cycloxaprid, a cis-nitromethylene neonicotinoid, represents a superior chemical alternative for managing imidacloprid-resistant *M. persicae*. Its distinct configuration confers not only higher acute toxicity but also, crucially, a more favorable ecotoxicological profile at sublethal levels. By significantly inhibiting the fecundity and suppressing the population growth parameters of resistant *M. persicae*, cycloxaprid exposure presents a low risk of inadvertently triggering pest resurgence, a common pitfall with some conventional insecticides. These combined attributes of high efficacy against resistant pests and population-suppressing sublethal effects position cycloxaprid as an ideal candidate for inclusion in integrated resistance management (IRM) strategies. To maximize its sustainable utility, its deployment should be strategically rotated with compounds from other, unrelated mode-of-action groups. Thus, cycloxaprid offers a potent tool not merely for immediate control, but for the sustainable, long-term management of resistant *M. persicae* populations in agricultural systems.

## Figures and Tables

**Table 1:** Toxicities of cycloxaprid and imidacloprid against *M. persicae* after 24 h.

Insecticides	Strain	Slope ± SE ^1^	LC_50_ (mg·L^−1^)	95% CI ^2^ (mg·L^−1^)	LC_25_ (mg·L^−1^)	95% CI ^2^ (mg·L^−1^)	*p*	RR ^3^
Cycloxaprid	field	1.55 ± 0.26	8.31	5.16–13.41	3.05	1.45–6.40	0.0198	3.65
	laboratory	1.76 ± 0.05	2.28	2.07–2.52	0.94	0.84–1.05	0.0001	1.00
Imidacloprid	field	1.22 ± 0.12	90.43	77.60–105.39	25.28	19.84–32.22	0.0091	15.97
	laboratory	1.61 ± 0.09	5.99	5.04–7.13	2.20	1.69–2.88	0.0001	1.00

^1^ SE, standard error. ^2^ 95% CI, 95% confidence interval. ^3^ RR, resistance ratio, LC_50_ of the field strain/LC_50_ of the susceptible strain.

**Table 2:** Field efficacies of cycloxaprid and imidacloprid to *M. persicae*.

Insecticide	Diluted Folds	AIC ^1^ (mg·L^−1^)	NAPA ^2^ (per Plot)	Efficacy % ^3^		
				3 Days	7 Days	14 Days
10% Imidacloprid WP ^4^	750	133.3	3273	40.57 ± 0.22 b	53.78 ± 0.69 b	55.24 ± 2.24 b
25% Cycloxaprid WP ^5^	2500	100.0	4280	93.33 ± 5.43 a	95.31 ± 3.95 a	84.79 ± 5.57 a

Means followed by a different letter are significantly different (*p* < 0.05 ANOVA). ^1^ AIC: Active ingredient concentration. ^2^ NAPA: Number of Aphids prior to application. ^3^ Survey results at different times after chemical treatment. ^4^ 10% Imidacloprid WP: A wettable powder formulation containing 10% (weight/weight) of the active ingredient imidacloprid. ^5^ 25% Cycloxaprid WP: A wettable powder formulation containing 25% (weight/weight) of the active ingredient cycloxaprid.

**Table 3:** Transgenerational effects of cycloxaprid and imidacloprid on developmental time and fecundity of the F_1_ generation of *M. persicae* when their parents (F_0_) were treated with the LC_25_ concentration of cycloxaprid and imidacloprid.

Variables	Control	Treatments (Mean ± SE ^4^)	
		Imidacloprid	Cycloxaprid
1st instar	1.932 ± 0.058 b	1.919 ± 0.057 b	2.190 ± 0.078 a
2nd instar	1.322 ± 0.070 b	1.622 ± 0.073 a	1.794 ± 0.093 a
3rd instar	1.678 ± 0.070 a	1.568 ± 0.064 a	1.651 ± 0.082 a
4th instar	1.797 ± 0.062 a	1.743 ± 0.061 a	1.905 ± 0.077 a
Adult (d)	24.136 ± 0.917 a	18.595 ± 0.826 b	21.158 ± 0.884 b
Mean longevity (d)	30.864 ± 0.919 a	25.446 ± 0.810 b	29.698 ± 0.862 a
Fecundity (offspring)	78.017 ± 1.828 a	63.716 ± 3.053 b	68.365 ± 2.604 b
APOP ^1^ (d)	0.728 ± 0.058 b	0.972 ± 0.070 a	0.825 ± 0.057 ab
TPOP ^2^ (d)	7.458 ± 0.103 c	7.808 ± 0.096 b	8.365 ± 0.117 a
GRR ^3^ (offspring/individual)	82.648 ± 1.467 a	75.032 ± 2.408 b	74.683 ± 2.300 b

Different letters within the same row suggest significant differences among control, LC_25_ concentrations of cycloxaprid and imidacloprid treatments. (at the *p* < 0.05, paired bootstrap test using TWOSEX MS chart program with 10,000 replications). ^1^ APOP: Adult pre-reproductive period. ^2^ TPOP: Total pre-reproductive period. ^3^ GRR: gross reproduction rate. ^4^ SE: Standard error.

**Table 4:** Transgenerational effects of cycloxaprid and imidacloprid on population parameters of the F_1_ generation of *M. persicae*.

Population Parameter	Control	Treatments (Mean ± SE ^1^)	
		Imidacloprid	Cycloxaprid
intrinsic rate of increase (*r_m_*) (d^−1^)	0.347 ± 0.004 a	0.323 ± 0.005 b	0.308 ± 0.004 c
Net reproduction rate (*R_i_*) (d^−1^)	78.017 ± 1.828 a	63.716 ± 3.053 b	68.365 ± 2.604 b
Mean generation time (*T*) (d)	12.532 ± 0.129 b	12.873 ± 0.132 b	13.722 ± 0.152 a
Finite rate of increase (*λ*) (d^−1^)	1.415 ± 0.006 a	1.381 ± 0.007 b	1.361 ± 0.006 c

Different letters within the same row indicate significant differences among control, LC_25_ concentrations of cycloxaprid and imidacloprid treatments. (at the *p* < 0.05, paired bootstrap test using TWOSEX MS chart program with 10,000 replications). ^1^ SE: Standard error.

## Data Availability

The raw data associated with this study are available from the corresponding author upon reasonable request.
